# Dietary interventions for modulating the gut microbiome in PCOS management

**DOI:** 10.3389/fendo.2026.1713408

**Published:** 2026-02-18

**Authors:** Ekta Patel

**Affiliations:** Osteopathic Medical Student (OMSII) Lake Erie College of Osteopathic Medicine, Erie, PA, United States

**Keywords:** chronic inflammation, diet intervention, gut dysbiosis, gut microbiome, PCOS (polycystic ovarian syndrome)

## Abstract

**Background:**

Polycystic ovary syndrome (PCOS) is a multifactorial endocrine disorder affecting about 10% of reproductive-age women. It is defined by insulin resistance, androgen excess, and chronic inflammation, which drive both reproductive and metabolic complications. Growing evidence suggests that gut microbiome dysbiosis contributes to PCOS by altering intestinal permeability, promoting endotoxemia, and worsening hormonal and metabolic dysfunction. Diet, as a modifiable factor, may offer a therapeutic route to restore microbial balance and improve outcomes.

**Objectives:**

This review aims to (1) synthesize evidence on how diet shapes gut microbiome composition in PCOS; (2) evaluate the effects of specific dietary patterns on microbial diversity, insulin sensitivity, lipid metabolism, and hormonal regulation; and (3) identify dietary components that may improve clinical outcomes.

**Methods:**

Evidence from observational studies, randomized trials, and meta-analyses was reviewed to assess how dietary interventions influence gut microbiome modulation and PCOS outcomes. Dietary patterns—including the Mediterranean diet, low-glycemic index diets, anti-inflammatory diets, time-restricted eating, and probiotic supplementation—were examined for their effects on microbiota and metabolic or hormonal measures.

**Results:**

Dietary interventions can beneficially alter gut microbiota, reduce systemic inflammation, improve insulin sensitivity, and lower androgen levels. The Mediterranean diet enhances microbial diversity and is associated with reduced PCOS risk. Low-glycemic index diets improve metabolic and hormonal profiles by lowering insulin demand. Anti-inflammatory diets and time-restricted eating may restore microbial rhythmicity and reduce inflammatory and endocrine imbalances. Probiotic supplementation, particularly with Lactobacillus and Bifidobacterium, strengthens gut integrity and benefits metabolic and hormonal outcomes. A multi-component dietary plan integrating high-fiber foods, probiotics, anti-inflammatory nutrients, low glycemic load, and structured eating patterns is proposed.

**Conclusion:**

Modulating the gut microbiome through diet is a promising, non-invasive, cost-effective strategy for PCOS management. By targeting insulin resistance, androgen excess, and inflammation, nutrition-based interventions can improve metabolic and reproductive outcomes. Long-term randomized trials are needed to strengthen causal evidence and guide personalized dietary approaches.

## Introduction

Polycystic ovarian syndrome (PCOS) affects about 1 in 10 women of reproductive age (1). According to the modified Rotterdam criteria, 2 out of these 3 symptoms are needed in order to have a diagnosis: evidence of oligo-anovulation such as irregular periods, high androgens (clinically or biochemically), or polycystic ovaries on ultrasound (2). Increased androgen in women results in excess facial and body hair, severe acne, and in extreme cases, can also cause male patterned baldness. Polycystic ovaries are characterized by small sacs of fluid, or cysts, that line the ovaries which contain immature eggs, ultimately resulting in failure to release the eggs. This in turn can cause irregular periods and difficulty getting pregnant. It is also notable that these symptoms are typically more severe in patients who suffer from obesity. Although PCOS is primarily known to be a reproductive disorder, it also has significant metabolic implications resulting in long term comorbidities such as gestational hypertension/diabetes, infertility, endometrial cancer, and a variety of metabolic diseases such as hypertriglyceridemia, hypercholesterolemia, and diabetes, increasing the risk of cardiovascular diseases (3). While the exact cause of PCOS remains unknown, chronic inflammation and insulin resistance are at the forefront as a driving mechanism.

The chronic inflammation seen in PCOS results from underlying immune and metabolic dysfunction and there is increasing evidence that implicates gut dysbiosis in the pathophysiology of PCOS as well. Studies have shown that an altered gut microbiome has an impact on gut inflammation, insulin resistance, and immunity (4). The gut microbiota consists of trillions of organisms which all have a symbiotic relationship and are crucial for maintenance of health as these microbes are involved in essential functions such as aiding in digestion, regulating the immune system and protecting the gut from harmful pathogens. Alterations of the microbial composition of the gut is called dysbiosis, and this dysbiosis results in metabolic and hormonal dysfunction (5, 6).

While it has been shown that androgens play a role in inflammation, metabolism, and homeostasis, there is also a link between the gut microbiome and androgen formation. The bacteria that reside in our gut metabolize estrogen into its unconjugated metabolites, returning it back into hepatic circulation in its active form. Once the composition of the bacteria in the gut is altered, the hormones in our body also become dysregulated and imbalanced (7). There has been an emerging theory linking the gut microbiome to PCOS. It has also been shown that the gut microbiome is highly affected by our diet, which is a modifiable factor (8). Emerging evidence suggests that gut microbiome dysbiosis directly contributes to the metabolic dysfunction seen in PCOS through several mechanisms. Altered microbial composition increases intestinal permeability and allows endotoxins such as lipopolysaccharide (LPS) to enter blood circulation triggering chronic systemic low-grade inflammation. This inflammatory state impairs insulin signaling, exacerbates insulin resistance, and stimulates excess androgen production in ovarian theca cells. Therefore, gut dysbiosis is not merely an associated finding but a mechanistic contributor to the metabolic and endocrine abnormalities characteristic of PCOS. This paper will focus on the effect of diet on microbial diversity and metabolic outcomes, specifically for PCOS.

## PCOS pathophysiology and chronic inflammation

Although the exact cause of PCOS is unknown, there are several factors that likely contribute, many of which are related to chronic inflammation. Adipose tissue excess is a well-known pro-inflammatory condition promotes chronic inflammation and insulin resistance. Increased insulin levels results in the increase of androgen production, which disrupts ovulation while increasing body and facial hair and acne. Insulin resistance can arise from multiple mechanisms including hormonal imbalance and inflammation of adipocytes (6). Key mediators of insulin resistance and chronic inflammation, are adipokines such as resistin, leptin, and adiponectin. The adipokine profile of increased adiponectin and decreased resistin and leptin has been associated with insulin resistance and PCOS, as this adipokine profile interferes with insulin signaling and enhances chronic inflammation due to hinderance of glucose absorption (6, 9). In some cases, hypermethylation of the CpG islands on the LAMIN gene causes reduced LMNA gene expression and disrupts insulin receptor signaling and glucose uptake (10). In other cases, dysregulated levels of microRNAs secreted by adipose tissue macrophages downregulate the Phosphoinositide 3-Kinase (PI3K)/Protein Kinase B (Akt)-Glucose Transporter Type 4 (GLUT4) signaling pathway in PCOS patients, which is essential in insulin signaling and glucose uptake. Evidence suggests that hyperinsulinemia that is a consequence of insulin resistance results in the inhibition of sex hormone binding protein which results in an increase in circulating free testosterone (11, 12). Additionally, insulin helps stimulate androgen synthesis in ovarian theca cells by upregulating 17-hydorxylase/17,20-lyase activity as well as by upregulating the steroidogenic acute regulatory protein (11). There is also increased 5-alpha reductase activity as a result of hyperinsulinemia which results in increased peripheral conversion of testosterone into the much more potent dihydrotestosterone which results in increased androgen symptoms like hirsutism and acne (13). These dysregulations not only increase androgens but also affect the way ovarian cells respond to insulin and therefore affect androgen production (3).

Chronic inflammation also results in increased concentrations of inflammatory cytokines, notably IL-1β, IL-6, TNF 2 receptor, and TNF-α (14, 15). Increased concentrations of these inflammatory cytokines can also result in the development of insulin resistance and imbalances in estrogen, progesterone, and androgens. IL -6 is a major proinflammatory cytokine as it regulates hepatic C-reactive protein secretion. In PCOS, IL-6 is upregulated due to insulin resistance, obesity, and dyslipidemia. IL-6 works in conjunction with TNF- α which acts on muscle, adipose tissue, and ovaries, further propagating the inflammatory response. Both IL-6 and TNF-α work to regulate steroidogenesis, granulosa and theca cell apoptosis, and follicular atresia Increases in these inflammatory markers can lead to androgen excess and ovulatory dysfunction seen in PCOS (16–19). TNF-α also propagates insulin resistance via interfering with insulin signaling by phosphorylating insulin receptor substrate 1 (IRS-1) and increasing hepatic glucose production by promoting lipolysis, secretion of free fatty acids, and increasing adiponectin (20–22). IL-1β and IL-18 have also been implicated in steroidogenesis along with oocyte maturation, however their implications in PCOS pathophysiology is not completely understood (23–26).

Although hyperandrogenism is a factor of PCOS, there is evidence that hyperandrogenism can also be a causative factor as well. Excess endogenous androgen production by the ovaries itself can also lead to the development of PCOS. The increase in concentration of androgens in addition to impaired androgen metabolism in PCOS results in chronic inflammation that causes follicular dysplasia, ultimately leading to ovarian dysfunction (27–31). Studies have also shown that hyperandrogenism in PCOS is directly related to diet induced inflammation (32). The interplay between gut dysbiosis and dietary factors involves an interconnected pathway in which the diet influences gut microbiome composition and the alterations in the gut microbiota promotes insulin resistance, chronic inflammation, and hormonal imbalances that ultimately drive PCOS development and progression. Dysregulation of the gut microbiome interferes with intestinal permeability, increasing the concentration of endotoxins such as LPS in the blood, activating the inflammatory process. When activated, there is decreased activation of insulin receptors through inhibitory serine phosphorylation, increasing insulin resistance (33).

## Pathophysiology of gut dysbiosis in PCOS

While the pathogenesis of PCOS is still unknown, there are possible mechanisms that explain the impact of the gut microbiome on PCOS. Lactobacillus and Bifidobacterium are two species of beneficial bacteria in our gut microbiome that help to decrease gut inflammation and the risk of infections while increasing insulin sensitivity, improving lipid metabolism, and increasing the number of short chain fatty acids (SCFAs). However, those who suffer with PCOS have been shown to have very low levels of these beneficial bacteria and instead have large amounts of harmful bacteria such as Shigella, Bacteroides, and Escherichia which produce large quantities of reactive oxygen species, a byproduct of cellular metabolism, as well as LPS, a lipopolysaccharide found on the outer layer of gram-negative bacteria which acts as an endotoxin. These factors can penetrate the gut wall and enter the bloodstream, causing endotoxemia and chronic inflammation. This occurs because once LPS has entered the bloodstream, the TLR4 pathway is activated, releasing inflammatory mediators and cytokines and increasing ROS production. One of the cytokines involved in this inflammatory pathway is IL-6, which also indirectly plays a role in androgen synthesis as it helps maintain testosterone activity by promoting the expression of the testosterone receptors (34). Testosterone levels are also further increased indirectly via the overstimulation of bile acid metabolism from the harmful bacteria in the gut. Therefore, inflammatory activation due to gut dysbiosis is connected to an increased production of androgens, including testosterone, as seen in PCOS (35, 36).

Bile acids also play multiple roles in regulating blood glucose levels. Firstly, they inhibit hepatic gluconeogenesis which promotes glycogen synthesis and insulin sensitivity. Hepatic glycogenesis is also promoted via bile acids as they activate PI3K/AKT/GSK3β signaling cascade, upregulating glycogen synthase expression. Bile acids also regulate blood glucose levels by enhancing glucose transporter 2 (GLUT2) function, which increases transport of glucose into hepatocytes leading to increased insulin production and secretion. Lastly, bile acids activate TGR5 which stimulates secretion of glucagon-like peptide-1 (GLP-1) promoting insulin production, delaying intestinal motility, inhibiting gastric emptying and acid secretion, and enhancing satiety leading to reduced food intake (37). Therefore, overstimulated bile acid metabolism can lead to insulin resistance, further exacerbating PCOS symptoms.When comparing a PCOS population to a healthy population, it was found that in the PCOS group, there was a marked decrease in the alpha diversity of the gut microbiome meaning that there was a marked decline in the health and diversity of the gut microbiome. There was also a change in the beta diversity meaning that there was a change in the variety of bacteria present as well. PCOS patients had reduced levels of Bacteroides, leading to dysregulation in bile acid metabolism, and an increase in *Firmicutes;* which has been linked to the development of obesity, type 2 diabetes, and metabolic syndrome which has been associated with PCOS. There was also significantly reduced levels of the beneficial bacteria, *Lactobacilli* and *Bifidobacteria* which enhance immunity and nutrient absorption. PCOS patients also had an increase in certain gram negative LPS producing bacteria such as *Faecalibacterium,Bifidobacterium, Blautia*, and *Escherichia/Shigella*, have been tied to the increased permeability of the intestinal barrier, leading to inflammation, insulin resistance, and obesity (35, 36, 39, 40).

The exact alterations in the gut microbiota in PCOS in unknown as each person’s microbiota is different, however the alterations in the gut regardless of the differences in composition from person to person all result in increased inflammation, impaired homeostasis and digestion, and alterations in immune responses which contribute to the progression of metabolic syndrome, obesity, and insulin resistance all of which are a component of the development and progression of PCOS.

## Role of diet in modulating the gut microbiome

The gut microbiome is composed of bacteria, viruses, fungi, and protozoa that all interact to create an ecosystem that work symbiotically to maintain a state of homeostasis. Alterations in the composition of the microbiota alters the homeostatic state of the body and has been shown to contribute to conditions/diseases (40). Throughout human life, the gut microbiota is influenced by multiple factors, however diet has an impact on the gut microbiota at any stage of life (41, 42). A healthy diet (such as the Mediterranean diet) has been shown to positively influence the composition of gut microbiome, whereas a ultra-processed diet or Western diet has been implicated in the propagation of diseases such as cardiovascular disease and obesity. The gut microbiome composition is not the same for any two people and there is no single composition that deems a gut microbiome as healthy; rather it is the ratios of good microorganisms to bad microorganisms and the interplay between diet, the immune system, and homeostasis of the gut that determines whether the composition of gut microbiota is healthy (43, 44). Therefore, it is essential to understand the impact of the different components of diet on the gut microbiota so that this information can be incorporated into practice to help promote a healthy state and prevents disease incidence and progression (44).

### Carbohydrates

There are two categories of carbohydrates, digestible carbohydrates (glucose, fructose, lactose, starch, etc.) and dietary fibers (inulin, glucans, etc.). The high sugar diet seen in the western diet has large amounts of digestible carbohydrates, which has been linked to significantly increased *Escherichia coli* levels in the digestive tract, causing gut inflammation, a widespread immune response, and increased levels of Akkermansia muciniphila (A. muciniphilia). A. muciniphila increases gut permeability by degrading the mucin layer and decreasing the production of SCFAs. In contrast, dietary fibers are vital in protecting the gut as its degradation into SCFAs improves insulin sensitivity and the barrier function of the gut. Some examples of good sources of dietary fibers are garlic, onions, chicory root, artichokes, bananas, and asparagus, as they contain inulin and oligofructose which are microbiota-accessible carbohydrates (MACs). MACs are important for the gut microbiome because they work as prebiotics, promoting the growth of healthy bacteria, such as Lactobacillus, which increase production of SCFAs, improving the gut barrier, increasing insulin sensitivity, and positively impacting lipid profiles (34).

### Proteins

Proteins can also be grouped into two categories, animal based and plant based. Animal based proteins, which are highly prevalent in the Western diet, have been correlated with an increase in harmful bile-tolerant anaerobic bacteria such as Bacteroides which, in high concentrations, are associated with increased gut inflammation. However, plant-based protein can increase levels of the beneficial bacteria like Lactobacillus and decrease levels of harmful bacteria like Bacteroides and Clostridium. Plant based proteins have been shown to be a good alternative to reduce gut inflammation-associated-proteins as they contain many resistant starches and fibers, which increase production of SCFAs and therefore gut barrier function (34).

### Fats

High fat diets also result in dysbiosis, causing insulin resistance, increased gut permeability, and systemic inflammation. A monounsaturated fat diet (one that includes pumpkin seeds, extra virgin olive oil, and peanuts) has been shown to increase gut microbiome diversity in both healthy individuals as well as individuals with metabolic syndromes. Diets rich in medium chain fatty acids (virgin coconut oil, human milk, and infant formula) increase growth of beneficial bacteria and help aid in weight loss and lipid catabolism by promoting microbial equilibrium and gut barrier integrity. Polyunsaturated fatty acids (sunflower oil, fatty fish, nuts, seeds) are considered essential fatty acids as our body cannot synthesize them, but they must be consumed in moderation, as a high ratio of omega-6 to omega 3 polyunsaturated fatty acids can lead to endotoxemia (34).

### Mediterranean diet

The Mediterranean diet has been deemed as one of the best diets to follow for promotion of health. This diet prioritizes consumption of unprocessed foods, vegetables, olive oil, and dairy products, moderate consumption of lean meats and fish and decreased consumption of red meats (45, 46). Multiple studies have shown the health benefits of a Mediterranean diet, however two studies have shown that this diet increases the concentration of health promoting bacteria Faecalibacterium prasunitizii and Roseburia spp., and decreased concentrations of Ruminococcus gnavus, Collinsella aerofaciens and Ruminococcus torques. The efficacy of the Mediterranean diet can be attributed to the production of SCFAs which improves insulin sensitivity and the barrier function of the gut, and promotes anti-inflammatory properties (45, 47, 48). The Mediterranean diet is primarily plant based and this type of diet is rich in polyphenols which are plant metabolites as well. These polyphenols interact with the gut microbiome and result in an increased concentration of health promoting and anti-inflammatory bacteria such as Bifidobacterium, Akkermansia, and Lactobacillus species (49–52).

### High fiber diet

There a many different types of fibers and the type of fiber impacts the gut microbiota in different ways, however, evidence indicates that a high fiber diet is correlated with an increase in two different beneficial bacteria, Lactobacillus spp. and Bifidobacterium species, both of which are able to digest more complex carbohydrates. This association has been seen in experiments comparing whole grain and wheat bran, with greater increases in the concentration of these two species in the group that was ingesting whole grains (53). Fibers are metabolized into simple or complex carbohydrates, and complex carbohydrates need to be metabolized further by the enzymes in the gut, however the concentration of these enzymes are limited and the body relies on the gut microbiota to ferment these complex carbohydrates into SCFAs which as stated above are pivotal in maintaining intestinal homeostasis and immune function (48). These polyphenols also regulate the production of SCFAs and bile acids both of which are integral in maintaining gut homeostasis.

### Ketogenic diet

The ketogenic diet is a high fat, low carbohydrate diet that has been used as a therapeutic intervention in the treatment of epilepsy. There has been emerging research indicating a therapeutic potential in the treatment of other conditions including obesity. Research has shown that the composition of the gut microbiota shifts as a result of the ketogenic diet with increases in Akkermansia, Lactobacillus, Roseburia, and Parabacteroides spp. and decreases in Turicibacter, Desulfovibrio, Escherichia, and Shigella species (54–56). The ketogenic diet has also been associated with decreases in proinflammatory intestinal T helper 17 (T_H_17) (57).

### Western diet

The Western diet is comprised of high calorie foods as well as ultra processed foods. This diet also primarily involved animal proteins, with less emphasis on fruits and vegetables. Studies show that the western diet is associated with decreased gut flora with Bacteroides predominance (58). SCFA production is decreased in this diet due to the lack of fiber intake, which results in decreased insulin sensitivity, decreased intestinal barrier functionality, and a pro-inflammatory state which can propagate multiple diseases/conditions (58). Food additives (non-nutritive artificial sweeteners, emulsifiers, etc.) are found in many ultra processed foods and can cause an increase in Bacteroides and Clostridium and a decrease in Bifidobacterium, which can cause glucose intolerance and insulin insensitivity. They can also cause bacterial translocation and systemic inflammation (59). Due to the chronic inflammatory state in patients who typically consume Western diets, there is a rise in diet-related chronic diseases including obesity and cardiovascular disease in those who consume this diet (60, 61).

## Review of evidence on different diets and PCOS

As demonstrated, the gut microbiome is greatly affected by our diets, so adjusting one’s diet can play a significant role in modulating the gut microbiome.

### Western diet

**Figure 1:**
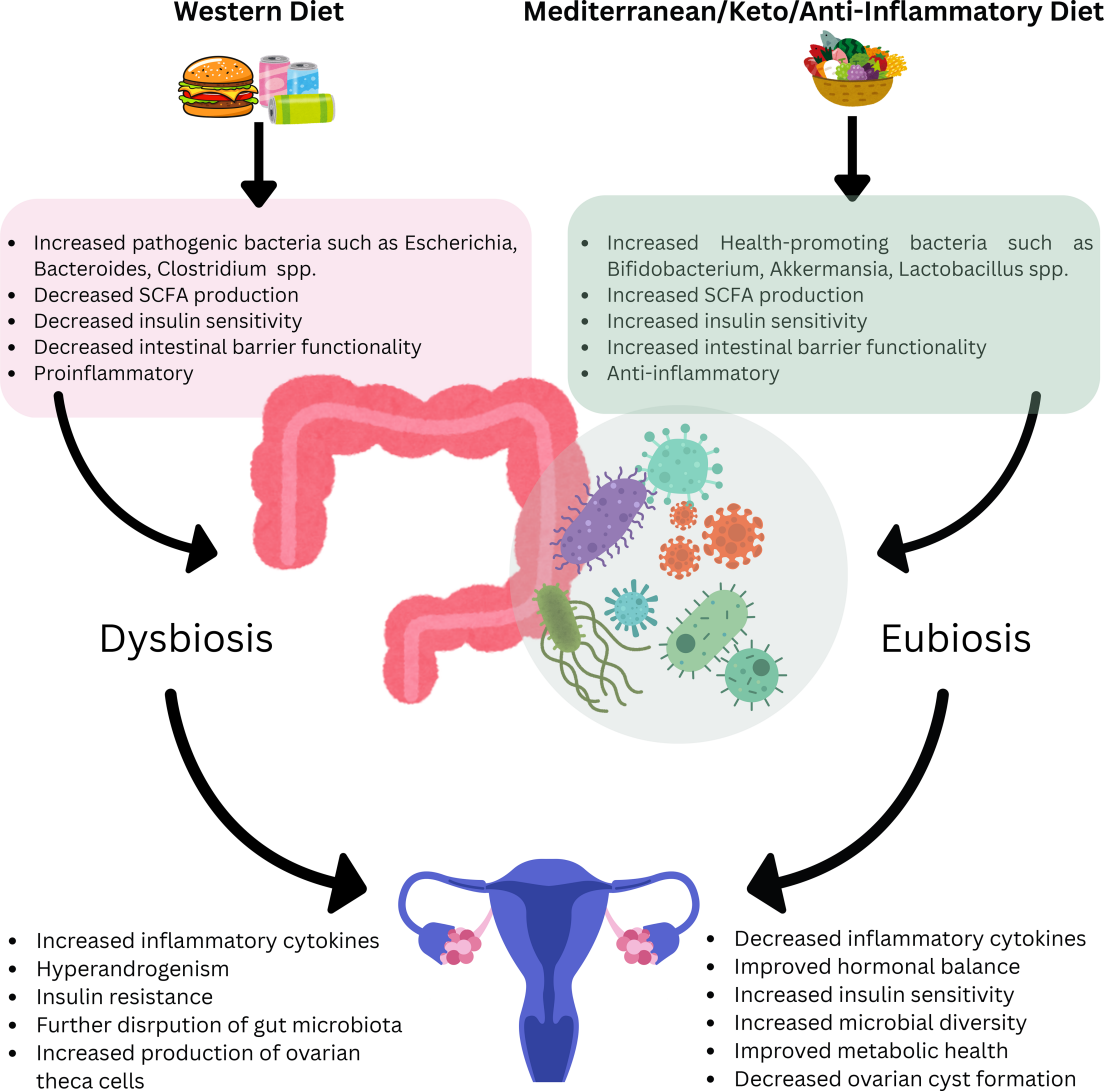
Schematic depicting diet and its impact on PCOS.

The Western diet, composed of an abundance of simple carbohydrates and saturated and trans fats is associated with conditions such as obesity and insulin resistance. Additionally the increased animal protein intake results in an increase in insulin-growth like factor which results in an increased production of ovarian-theca cells, predisposing patients to PCOS (62). The Western diet is also associated with disruption of the intestinal microbiota which results in chronic inflammation as well as hyperandrogenism and insulin resistance (63). A high fat high sugar diet is also associated with the metabolic impairment as well as the hyperandrogenism/elevated testosterone as seen in PCOS pathophysiology, and this elevation in testosterone is associated with ovarian cyst formation (**Figure 1**) (64).

### Mediterranean diet

One diet that could be further studied in order to help treat PCOS symptoms and potentially address the root cause of PCOS, is the Mediterranean diet. The Mediterranean diet consists of high fiber intake (such as whole grains, fruits, vegetables, legumes), olive oil, and nuts. As written above, a combination of these ingredients could positively impact the diversity of the gut microbiome and help restore gut integrity as well as increase insulin sensitivity and significantly lower lipid profiles. Findings from a study performed by Ajorlouie et al. showed that high adherence to a Mediterranean diet significantly decreased the odds of women developing PCOS as the diet led to improved hormonal balance as well and increased microbial diversity. The study showed there was a 32% reduction in risk in developing PCOS if the patient strictly maintained a Mediterranean diet after accounting for potential confounding factors such as age, body mass index, physical activity, and total energy intake. The researchers also had multiple groups in order to test if adherence to the diet has had an effect on PCOS outcomes. After adjusting for the same confounding factors, the researchers found that those who closely followed the diet had a 43% reduced risk in developing PCOS (**Figure 1**) (65).

### Ketogenic diet

Another diet which has been posited to positively impact those with PCOS is the ketogenic diet; a popular diet that has a high potential to delay aging and encourage fat loss. This diet consists of consuming about 20-50g of carbohydrates in a day while consuming a greater amount of healthy fats and proteins at a level that is calculated using the patient’s ideal body weight. A 2025 study by Fleigle et al. showed that a ketogenic diet can be a beneficial aid in improving symptoms such as irregular menstruation, infertility, weight gain and ovarian hyperstimulation syndrome by improving biochemical measures such as levels of androgens, lipids, blood glucose, and insulin, along with decreasing insulin resistance, and improving the LF/FSH ratio. This diet may be effective in modulating PCOS symptoms as decreasing the amount of carbohydrates in the body causes a switch from glucose metabolism to ketone metabolism which creates ketone bodies. This leads to the inhibition of ghrelin and cerebral neuropeptide Y release, hormones that are released in a hunger state. Reducing carbohydrate intake has a positive impact on the hypothalamic-pituitary- ovarian axis as there is reduced levels of LH effectively increasing insulin sensitivity and decreasing androgen stimulation, lessening the severity and prevalence PCOS symptoms (66).

### Low carbohydrate diet

The low carbohydrate/low glycemic index diet can be an explored diet as high sugar diets have been linked to insulin resistance and inflammation. A meta-analysis of randomized controlled trials showed that a low carbohydrate diet lead to significant improvements in not only BMI (especially in overweight individuals), with a standardized mean difference of -1.04, but also in insulin resistance, LDL and total cholesterol, and testosterone with standardized mean differences of -0.66, -0.67, and -1.79, respectively (67). This diet would help treat PCOS symptoms as it would improve insulin sensitivity through a reduction in insulin demand, which can indirectly help lower testosterone levels. This diet would also help lower inflammatory markers and decrease chronic inflammation through a reduction in refined carbohydrates in the diet.

### Anti-inflammatory diet

An anti-inflammatory diet could help combat PCOS symptoms as the ovarian cysts are a result of chronic inflammation. An anti-inflammatory diet is one that focuses on increasing intake of foods that are rich in antioxidants, such as whole minimally processed foods, focusing on plant-based foods and healthy fats. A study conducted by Chi et al. highlighted how a diet rich in phytochemicals (known for their anti-inflammatory properties) can be beneficial in the management of PCOS as the incorporation of these foods have had a positive effect on metabolic health as well as hormonal profiles. The anti-inflammatory diet lowered fasting blood sugar, fasting insulin, triglycerides, total cholesterol, as well as low density lipoprotein (LDL). It also lowered total testosterone levels, LH/FSH ratio, DHEA-S, and increased sex hormone binding globulin (SHBG) (68).

### Time-restricted eating

Another interesting diet model is time restricted eating, as the gut microbiome thrives on predictable rhythmic oscillations, akin to our circadian rhythm. Studies have shown that with time restricted eating, beneficial bacteria such as Lactobacillus were able to be reinstated and cause gut eubiosis (69). A study by Feyzioglu et al. showed that a 6-week program of eight-hour time-restricted feeding resulted in major improvements in not only anthropometric and metabolic profiles, but also in hormonal profiles as well (70).

### Probiotic supplementation

Lastly, supplementation with probiotics has shown to increase the diversity of the gut microbiome, specifically reimplementing strains of Lactobacillus and Bifidobacterium, leading to beneficial changes in metabolic profiles as well as hormone levels. Modulating the gut microbiome positively influences bile acid metabolism which in turn modifies glucose and lipid metabolism through the FXR and TGR5 receptors. There has also been evidence that these strains of bacteria help decrease testosterone levels by improving insulin resistance, therefore stabilizing insulin levels. Probiotics have also shown to increase SCFA production, which helps fight against insulin resistance by strengthening the mucosal barrier and preventing endotoxemia. They also have anti-inflammatory properties as it decreases proinflammatory molecules such as TNF-a and IL-6 (71).

### Comparative analysis of dietary interventions

While the Mediterranean, low glycemic, anti-inflammatory, and ketogenic diets all demonstrate benefits for PCOS, their mechanisms differ. The Mediterranean diet appears most consistently associated with improved microbial diversity and increased SCFA production due to its high fiber and polyphenol content. Low-glycemic diets primarily improve insulin resistance through reduced postprandial glucose excursions, but their impact on microbial diversity is less pronounced. Anti-inflammatory diets reduce systemic inflammation and modulate microbial composition through antioxidant-rich foods but may be less effective than Mediterranean dietary patterns in increasing beneficial bacterial such as *Lactobacillus* and *Bifidobacterium*. Time restricted eating impacts microbial rhythmicity rather than composition alone. Together, these comparisons highlight that while each dietary pattern offers unique benefits, the Mediterranean diet currently has the strongest evidence for holistic gut-microbiome-endocrine improvements in PCOS.

Taken together, these findings demonstrate that dietary interventions influence PCOS development and progression through distinct but overlapping microbiome-mediated mechanisms. While the magnitude of effects varies by dietary pattern, consistent improvements in insulin sensitivity, inflammatory markers, and androgen profiles are observed across several interventions. A comparative summary of dietary patterns, representative studies, microbiome alterations, and PCOS- related outcomes is presented in **Table 1**.

**Table 1:** Dietary interventions, gut microbiome modulation, and metabolic-endocrine outcomes in PCOS.

Dietary intervention	Studies	Gut microbiome effects	Proposed microbiome-mediated mechanisms	Metabolic outcomes	Hormonal/PCOS- related outcomes
Mediterranean Diet	Ghosh et al., 2020 (45); Wang et al., 2021 (46); Ajorlouie et al., 2025 (65)	↑ *Faecalibacterium prausnitzii*, *Roseburia* spp.; ↑ microbial diversity; ↑ SCFA production	SCFA-mediated enhancement of gut barrier integrity, reduced endotoxemia, improved insulin signaling	↓ insulin resistance, ↓ dyslipidemia, improved glucose metabolism	↓ PCOS risk (32–43%), improved hormonal balance
High-Fiber Diet	Costabile et al., 2008 (53); Zhang, 2022 (8)	↑ *Lactobacillus* spp., *Bifidobacterium* spp.; ↑ SCFAs	Fermentation of microbiota-accessible carbohydrates improves immune regulation and intestinal permeability	↑ insulin sensitivity, ↓ systemic inflammation	Indirect improvement in hyperandrogenism via improved insulin signaling
Ketogenic Diet	Olson et al., 2018 (54); Ang et al., 2020 (57); Fleigle et al., 2025 (66)	↑ *Akkermansia*, *Lactobacillus*, *Roseburia*; ↓ *Escherichia*, *Shigella*	Reduced carbohydrate availability lowers insulin demand; suppression of Th17-mediated intestinal inflammation	↓ insulin resistance, ↓ blood glucose, ↓ lipid levels	↓ total testosterone, improved LH/FSH ratio, improved menstrual regularity
Low-Carbohydrate/Low-Glycemic Index Diet	Zhang et al., 2019 (67)	Modest shifts toward beneficial taxa; reduced dysbiosis	Reduced postprandial glucose excursions decrease hyperinsulinemia	↓ BMI, ↓ LDL cholesterol, ↓ insulin resistance	↓ circulating testosterone levels
Anti-Inflammatory Diet	Chi et al., 2024 (68)	↑ anti-inflammatory bacterial taxa; improved microbial balance	Phytochemical-mediated reduction in oxidative stress and pro-inflammatory cytokines	↓ fasting glucose, ↓ insulin, ↓ triglycerides	↓ testosterone, ↓ LH/FSH ratio, ↑ SHBG
Time-Restricted Eating	Xia et al., 2023 (69); Feyzioglu et al., 2023 (70)	Restoration of microbial circadian rhythmicity; ↑ *Lactobacillus*	Alignment of feeding–fasting cycles with microbial oscillations improves metabolic signaling	↓ body weight, improved metabolic parameters	Improved menstrual regularity and hormonal profiles
Probiotic Supplementation	Guevara et al., 2024 (71); Hanna et al., 2025 (38)	↑ *Lactobacillus* spp., *Bifidobacterium* spp.; ↑ SCFA production	Modulation of bile acid metabolism via FXR/TGR5 signaling; reduced endotoxemia	↓ insulin resistance, ↓ inflammatory cytokines	↓ androgen levels, improved metabolic and hormonal profiles
Western Diet	Christ et al., 2019 (60); Roberts et al., 2017 (64)	↓ microbial diversity; ↑ LPS-producing *Bacteroides*, *Escherichia*, *Shigella*	Increased intestinal permeability, endotoxemia, and chronic low-grade inflammation	↑ insulin resistance, ↑ obesity risk	↑ hyperandrogenism, ovarian cyst formation

up arrow (↑) = increase in

down arrow (↓) = decrease in

## Proposed treatment plan: dietary intervention

### Baseline assessment

Firstly, after a diagnosis of PCOS is established, a baseline assessment would be necessary in order to obtain a holistic understanding of the patients’ symptoms and to have data to act as a reference point. There are many factors that must be measured to consider all proposed causes of PCOS symptoms into account. This can be done through a gut microbiome analysis via shot gun analysis of stool samples (72), blood tests for metabolic markers (fasting insulin and glucose levels, lipid panels, etc.) and hormone levels (FSH, LH, total testosterone, DHEA-S) as well as a dietary assessment.

### Intervention components

Together, these findings suggest that while multiple dietary patterns such as Mediterranean, low-glycemic index, ketogenic, and anti-inflammatory diets, demonstrate benefits for PCOS, overlapping components allow them to be synthesized into a cohesive therapeutic pattern, with the help of dieticians and nutritionists. The Mediterranean-style diet currently has the strongest evidence for enhancing microbial diversity and improving metabolic outcomes, while low-glycemic approaches may be particularly effective for reducing insulin demand. Anti-inflammatory diets complement these by reducing oxidative stress and cytokine production, and time-restricted eating uniquely contributes by restoring microbial circadian oscillations (1, 73).

### Monitoring and evaluation

As it could potentially take months for eubiosis of the gut microbiome, PCOS patients should be monitored and evaluated once a week for about 12–16 weeks. Some changes that should be tracked are hormonal symptoms (acne, menstrual cycles, and body and facial hair), metabolic markers, and subjective gut health such as bloating, bowel movements/habits, etc. Probiotic supplementation should be administered for a minimum of 8–12 weeks at doses of 10^9–^10^10^ CFU/day, preferably with multi-strain formulations containing *Lactobacillus* and *Bifidobacterium* (2). Dietary interventions should be evaluated at baseline, 6 weeks, and 12 weeks for changes in metabolic markers, hormonal profiles, gastrointestinal symptoms, and adherence. Time-restricted eating protocols typically use 8-10- hour food windows, and patients should be monitored for tolerability, menstrual changes, and hypoglycemia risk.

## Discussion

PCOS is a reproductive, endocrine, and metabolic syndrome that requires certain diagnostic criteria to be met to be diagnosed, however, the exact pathophysiology and the mechanisms behind the development and progression is still unclear. Given the metabolic changes seen in PCOS, many studies have been conducted to try and elucidate a causal link between diet and PCOS. Evidence suggests that diet may propagate the development but also the progression of PCOS, specifically due to alterations in the gut microbiota and the chronic inflammation that results.

Chronic inflammation contributes to insulin resistance through multiple mechanisms, including altered adipokine signaling, epigenetic disruption of insulin receptor pathways via gene hypermethylation and microRNA regulation, and increased production of pro-inflammatory cytokines. These inflammatory mediators further exacerbate insulin resistance by promoting hormonal imbalances (6, 9–12). The insulin resistance stimulates endogenous androgen synthesis as well as promotes peripheral conversion of testosterone into dihydrotestosterone which promotes the androgenic symptoms of PCOS (11, 13). Increased concentrations of IL-1β, IL-6, TNF 2 receptor, and TNF-α results in hormonal imbalances, insulin resistance, obesity, and dyslipidemia all of which are factors involved in PCOS (14–19). Alterations in the gut flora can further propagate chronic inflammation and has been implicated in the pathophysiology of PCOS, although the exact mechanisms are not completely understood.

The gut flora is composed of trillions of organisms that typically work in a symbiotic relationship to maintain homeostasis. Alterations of the normal gut flora, or dysbiosis, can result in numerous different types of conditions/diseases. Emerging evidence indicates that dysbiosis can be a precipitant or contributing factor to the development and progression of PCOS. The type of diet a person follows can also be a contributing factor as diet impacts the gut microbiota as well. The most common alterations in the gut flora are increasing concentrations of E. coli, Bacteriodies, Shigella, and Akkermansia spp. The increase in these species results in an environment that is proinflammatory. The alterations in the flora of the gut results in chronic inflammation and this chronic inflammation has been implicated as a potential pathogenic factor in the development and progression of PCOS. Studies have shown that different diets have differing implications on the gut microbiota and dome diets can be used as a therapeutic intervention in the treatment of PCOS. The western diet has been widely studied as a pro-inflammatory diet, with well-established associations between this diet type and obesity and other cardiovascular diseases (58–64). The Western diet has also been associated with hyperandrogenism and the development of polycystic ovaries, two of the three Rotterdam criteria (2–6, 58–64). On the other hand, diets that prioritize a well-balanced meal with more fruits, vegetables, and whole grains such as the Mediterranean or Anti-Inflammatory diet have been shown to have a hormone balancing effect and positive effect on glucose balance which decreases insulin resistance. There is limited evidence regarding the effect of different diet types on decreasing PCOS incidence, however the impacts of these diets on the gut microbiota and on chronic inflammation show therapeutic promise. Further research with large, randomized control trials and dietary standardization is necessary to establish a substantial link between dietary modification and PCOS incidence and progression.

## Future directions and research gaps

Continued research regarding the link between PCOS and the gut microbiome is necessary in order to provide concrete evidence on if stabilizing the gut microbiome could help treat PCOS symptoms and help move closer to finding a cure. There are still many gaps in the research which could be closed with long-term randomized controlled trials. It would be interesting to work with nutrition specialists or registered dietitians to determine if there is a link between the use of personalized nutrition based on microbiome composition in treating PCOS. Lastly, it should be considered that diet may not be the sole answer; other areas of interest include the effect of lifestyle changes such as exercising and changing sleep patterns on PCOS and the integration of insulin-sensitizing medications on not only PCOS but also the gut microbiome.

Future work should explore the role of personalized nutrition based on individual microbial signatures, metabolic profiles, and genetic predispositions. Integrating dietary interventions with genomic and metabolomic analyses may allow for precision nutrition tailored to each PCOS patient. This will require multidisciplinary collaboration among dietitians, endocrinologists, microbiologists, and data scientistic to develop clinical tools that translate microbiome data into actionable dietary plans.

Current research is limited by short study durations, small sample sizes, lack of dietary standardization, and inconsistent definitions of PCOS phenotypes. Most trials do not exceed 8–12 weeks, making it difficult to determine long-term sustainability, adherence challenges, or potential unintended effects of restrictive diets such as ketogenic or time-redistricted patterns. Translating dietary interventions into clinical practices is further complicated by variability in patient access, socioeconomical factors, cultural food preferences, and lack of consensus on optimal dietary dosing. Larger, long-term randomized controlled trials are needed to strengthen casual inference, determined durability of microbiome changes, and evaluate real-work feasibility.

## Conclusion

The first step in managing PCOS may be modulating the gut microbiome. Diet can serve as a readily available approach to rebalancing the gut microbiome in a non-invasive cost- effective manner. Proper dietary interventions could be the future for PCOS treatment as it not only improves hormonal, metabolic, and inflammatory profiles but also systemic health through improvement in weight and insulin resistance. These interventions could also help protect fertility in those who suffer from PCOS. With continued research, precision nutrition/medicine could become the answer to PCOS management and help bring us closer to curing PCOS.
